# Attitude towards sports, psychological resilience and life engagement: a study on earthquake victims

**DOI:** 10.3389/fpubh.2026.1844066

**Published:** 2026-07-02

**Authors:** Tolga Tek, Arif Özsarı, Murat Tilki, Şekip Can Deli, M. Çağrı Çetin

**Affiliations:** 1 Faculty of Sport Sciences, Selcuk University, Konya, Türkiye; 2 Faculty of Sport Sciences, Mersin University, Mersin, Türkiye; 3 Institute of Social Sciences, Mus Alparslan University, Mus, Türkiye

**Keywords:** attitude towards sports, earthquake victims, life engagement, psychological resilience, sports

## Abstract

**Purpose:**

This investigation examines associations among sporting attitudes, psychological resilience, and life engagement in children and adolescents participating in aquatic training programs following earthquake exposure.

**Method:**

The study employed a correlational survey design involving 124 earthquake survivors (Age M = 12.69; SD = 3.34), comprising 72 females (58.1%) and 52 males (41.9%) enrolled in swimming training programs in Hatay province. The present investigation employed a correlational survey methodology to examine the relationships between study variables.

**Research data:**

The data collection tools consisted of the Personal Information Form, the Attitude Toward Sports Scale, the Child and Adolescent Psychological Resilience Scale, and the Life Commitment Scale. Internal consistency reliability was assessed using Cronbach’s Alpha (*α*) coefficients to evaluate the psychometric integrity of the scales. In the scale scoring procedures, the average scores obtained from the component items for each measurement tool were used. Statistical analyses encompassed descriptive statistics, correlation analyses, and multiple regression modeling within the correlational research framework.

**Result:**

Correlation analyses revealed a significant positive moderate association between interest in sports and psychological resilience (*r* =0.391), and a significant positive weak association between interest in sports and life engagement (*r* = 0.252); a significant positive weak relationship between sport lifestyle integration and psychological resilience (*r* = 0.242); and a significant positive weak relationship between active sport participation and life engagement (*r* = 0.183). Multiple regression analyses demonstrated that interest in sports significantly predicted both psychological resilience (*β* =0.473) and life engagement (*β* = 0.357).

**Conclusion:**

Individuals experiencing major traumatic events, particularly seismic disaster survivors, may benefit from structured sport-based interventions, toward sporting activities to enhance psychological resilience capacities and life engagement outcomes.

## 1 Introduction

**Figure 1:**
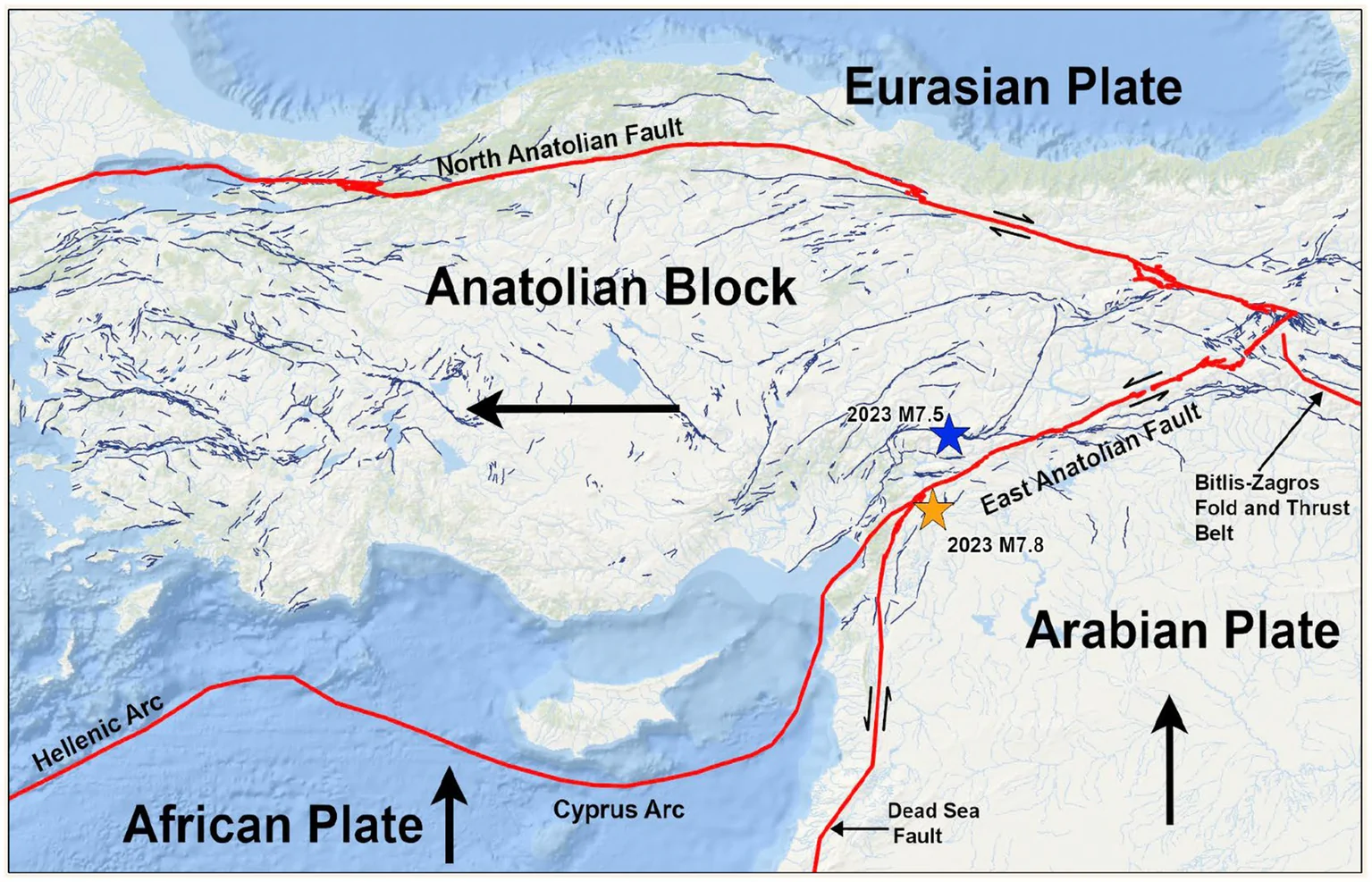
Tectonic setting map of the February 6, 2023 Kahramanmaraş Earthquakes. From tectonic setting [Map], by (62) (https://earthquake.usgs.gov/storymap/index-turkey2023.html). In the public domain.

On 6 February 2023, Turkey experienced one of its most devastating seismic events in recent history the Kahramanmaraş earthquake which profoundly impacted 11 provinces across the nation: Adana, Adıyaman, Diyarbakır, Elazığ, Gaziantep, Hatay, Kahramanmaraş, Kilis, Malatya, Osmaniye, and Şanlıurfa. This catastrophic natural disaster resulted in extensive human casualties, with 107.204 individuals sustaining injuries and 53.537 fatalities recorded (1) (Figure 1).

**Figure 2:**
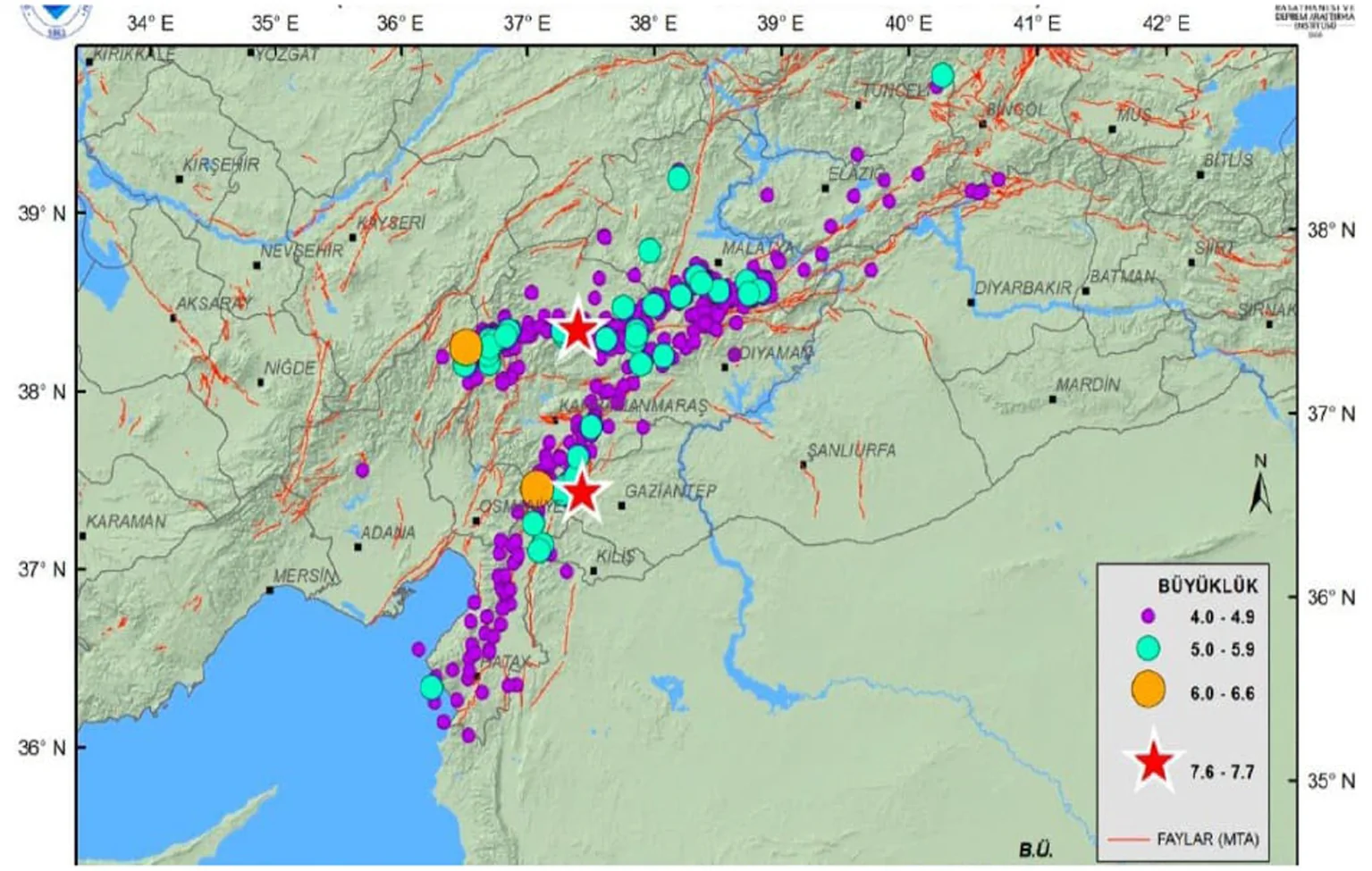
Earthquake activity map of Türkiye and surrounding regions in 2023. The map illustrates seismic activity recorded between January 1, 2023, and December 31, 2023, totaling 58,881 events. Adapted from *2023 Yılı Deprem Harita, Grafik ve Tabloları*, by Boğaziçi University Kandilli Observatory and Earthquake Research Institute (63) (http://www.koeri.boun.edu.tr/sismo/2/deprem-verileri/yillik-deprem-haritalari/2023-yili-deprem-harita-grafik-ve-tablolari/). Copyright 2024 by KRDAE.

This earthquake reshaped daily life far beyond its immediate impact zone (2). The province of Hatay, which serves as the geographical focus of this investigation, ranks among the most severely affected regions following this seismic catastrophe. Natural disasters, particularly earthquakes of such magnitude, necessitate prioritizing vulnerable populations in protective intervention strategies, with children representing the demographic requiring immediate and sustained attention. Young individuals experience profound psychological trauma when confronted with extraordinary disruptions to their established daily routines and environmental stability. The psychological sequelae of such catastrophic events frequently manifest as enduring mental health challenges that resist rapid resolution, commonly precipitating post-traumatic stress disorder and associated psychological complications throughout the recovery trajectory (3) (Figure 2).

The devastating consequences of seismic events extend beyond immediate physical destruction, fundamentally compromising both individual and collective psychological well-being. Survivors frequently develop a constellation of mental health disorders, including depressive episodes, anxiety syndromes, and acute stress reactions (4). It should not be forgotten that if security measures are not taken after major and destructive earthquakes, social problems can also create crises. With the destruction of the structures that concern society, education systems, health conditions and interpersonal relationships can be deeply negatively affected. In these times, plans should be presented specifically for each earthquake victim and action should be taken. In order to move from the crisis situation to rapid normalization, the idea of reducing the damage with additional management strategies should be taken as a basis (5). On the other hand, it is thought that reliable educational integration in the implementation of these plans and strategies will benefit post-earthquake recovery practices in the crisis region (6, 7). The implementation of social support frameworks appears to be a critical intervention strategy in post-disaster recovery contexts (8). Preserving pre-earthquake daily routines could prove a fundamental mechanism for mitigating anxiety responses among children. When cognitive resources are redirected through engaging recreational activities and structured play interventions, the preoccupation with traumatic earthquake-related cognitions demonstrates measurable reduction. Furthermore, the facilitation of activities designed to expand and strengthen social networks promotes interpersonal communication competencies. Such interventions provide essential protection against the debilitating psychological and traumatic sequelae associated with seismic disasters (9). Earthquakes, as a common reality affecting people of all ages, cause traumas that put people under great stress. Comprehensive disaster response efforts are critical as engaging in spiritual healing activities for earthquake victims increases their resilience and enables their lives to return to normal quickly (10, 11). Within this framework, the integration of complementary methodologies incorporating structured sports-based interventions represents a particularly promising approach for bolstering psychological resilience among pediatric and adolescent populations in post-disaster settings, thus facilitating the restoration of life engagement to pre-earthquake baseline levels.

The inherent competitive dynamics and stress-adaptive mechanisms embedded within athletic participation serve as catalysts for strengthening both physiological and psychological adaptive capacities. The exploitation of such sport-inherent restorative characteristics represents a critical avenue for facilitating expedited post-disaster life reintegration processes. Expert-supervised athletic engagement during seismic crisis periods constitutes an essential component of accelerated recovery pathways for disaster-affected populations (12). Empirical evidence demonstrates that young disaster survivors participating in recreational sporting activities exhibit enhanced motivational profiles, increased relaxation responses, and substantive improvements in future-oriented optimism (13). In addition, individuals who are interested in sports in various aspects may want the specialist who takes care of them in the sports environment to encourage them by directing them. These positive notifications and a positive climate environment have a significant impact on developing individuals interested in sports (14). Together with the motivation provided by engaging in physical activity after natural disasters, it plays a critical role in accelerating recovery in the post-traumatic period (15). The post-2011 Christchurch earthquake hosting of the Cricket World Cup exemplifies the transformative power of major sporting events in disaster-affected communities. This strategic deployment of high-profile athletic competition generated significant morale enhancement among regional populations while simultaneously catalyzing rapid sports infrastructure restoration and accelerated urban regeneration processes (16). This empirical case study illustrates the profound impact achievable through positive sport-oriented attitudes at both individual and policy-making levels. Contemporary disaster management frameworks necessitate the systematic integration and amplification of sporting events as core recovery components. Enhanced research investment examining sport-based contributions to recovery processes remains critically needed, as such investigations will increasingly illuminate wide range of societal benefits generated by robust sporting ecosystems (17).

The quantification of sport attitude contributions to psychological resilience enhancement and life engagement restoration enables precise calibration of their inclusion within strategic recovery planning frameworks. Sport participation provides adolescent populations with opportunities to transcend life adversities, facilitating success-oriented experiences and resilient behavioral development (18). These developmental cohorts possess inherent adaptability prospects for problem-resolution across life domains. The cultivation of emotion regulation mechanisms during high-stress situations enables effective crisis navigation (19). Individuals’ potential can be transformed into skills if properly guided (20). Positive sport attitude development during post-earthquake chaos periods theoretically accelerates normalization process completion, suggesting significant corrective utility for athletic engagement protocols in disaster recovery contexts.

Representing recurrent manifestations of natural geological processes, large seismic events like earthquakes generate profound material and existential disruptions that directly compromise human wellbeing across multiple dimensions, although the physical and psychological symptomatology observed among earthquake survivors can be substantially ameliorated through expedited interventions implemented by specialized multidisciplinary teams within appropriate institutional frameworks (21). Even though the review of literature indicates substantial scholarly attention devoted to psychological recovery processes and life adaptation mechanisms following seismic disasters (22), systematic examination of sport-based interventions within these recovery paradigms still remains underrepresented in previous studies. In particular, there is limited research on the potential relationship between sports activity-based approaches and psychological resilience and life satisfaction within the scope of attitudes toward sports. The present investigation, therefore, addresses this identified literature gap by formulating specific hypotheses that systematically examine ameliorative methodologies that can be employed to mitigate earthquake-induced traumatic effects among pediatric and adolescent athletic populations, specifically focusing on sport attitude mechanisms as mediating variables.

Attitudinal constructs encompass multidimensional cognitive-affective frameworks comprising individual perspectives, behavioral predispositions, perceptual orientations, evaluative judgments, and emotional responses directed toward specific activities, circumstances, or entities. These complex psychological formations emerge as dynamic reflections of intricate interactions between psychological states, social contextual factors, and environmental determinants (23). A positive attitude towards sports can generate multifaceted benefits across health optimization, performance enhancement, and psychosocial developmental domains. In contrast, negative attitudes, maladaptive behavioral patterns, and trauma-induced psychological disruptions necessitate interventions incorporating both supportive and preventive methodological approaches during periods of psychological vulnerability (24). Sport-directed attitudes may exert a profound influence on several life domains, extending from interpersonal social interaction patterns to personal development trajectories. These attitudinal constructs could foster both mental cognitive architecture and physical developmental structures while maintaining robust interconnections with self-efficacy belief systems and broader psychosocial maturation processes (25).

Natural disasters can exert debilitating effects on individuals, often manifesting as significant psychological distress. As Wu et al. (26) highlight, traumatic and stressful events can disrupt brain function and precipitate psychological disorders, including depression and post-traumatic stress disorder. In such contexts, psychological resilience emerges as a crucial protective factor. This construct refers to the capacity to maintain healthy psychological functioning when confronted with adversity. Exposure to shocking and stressful events can induce a spectrum of mental health challenges, ranging from mild disturbances to severe pathologies. Thus, it is imperative to comprehend immediate reactions, implement effective interventions, and establish robust support systems (27). Psychological resilience can be conceptualized as the ability to adapt and remain flexible in the face of difficult circumstances, thereby mitigating psychosocial consequences and mental health disorders (28). Psychological resilience is a multifaceted construct that not only enhances performance but also fosters the development of essential life skills (29). Beyond facilitating an individual’s ability to cope with diverse life challenges, psychological resilience functions as a catalyst for personal growth, cultivating a positive outlook grounded in internal consistency and elevated awareness (30). This resilient mindset, in turn, can significantly bolster an individual’s life engagement.

Life engagement has garnered considerable scientific interest in recent years. It often embodies active participation in purposeful and meaningful activities, which improves an individual’s sense of well-being and satisfaction (31). On top of that, an individual’s propensity for optimism, a broad social network, and the capacity for emotional expression are all factors that enhance their commitment to life (32). Following an earthquake, identifying and planning for apparent risks can yield vital data for the formulation of effective health policies (33). Through the multifaceted and inclusive framework of social service provisions, access to essential services and resources such as sports activities, rehabilitation programs, and psychosocial support can be facilitated for earthquake survivors during the arduous recovery process (34). In contemporary public health, a diverse array of research fields has emerged, primarily focused on mitigating threats to collective well-being. Despite sport’s considerable promise to address numerous public health issues, its full capacity remains underutilized. However, the increasing integration of sports-based initiatives into public health efforts underscores its strong facility as a tool for community development (35).

Natural disasters, such as earthquakes, are widely recognized for their profound impact, leading to a range of physical and psychological challenges for affected individuals (3, 4). It has been observed that earthquake-related research primarily focuses on construction, urban consolidation planning, and general areas of interest to society, and the scarcity of studies on the role of sports in the difficult post-earthquake process draws attention (5). Given the established rehabilitative potency inherent in athletic engagement, sport-based interventions emerge as viable restorative tools capable of achieving partial or complete easing of such adverse effects. This study was, therefore, designed to examine the relational dynamics between attitudes toward sports, psychological resilience, and life engagement in children and adolescents who participated in swimming training programs within earthquake-affected regions. The research was conducted in Hatay Province, a region severely impacted by the recent earthquake. We anticipate that the findings will not only enrich existing literature but also offer significant contributions to countries affected by natural disasters (both past and future), as well as to policymakers, educators, and parents in these nations.

In light of these considerations, we propose the following six hypotheses:

*Hypothesis* 1: Interest in sports, a subdimension of attitude towards sports, positively influences psychological resilience.

*Hypothesis* 2: Sport lifestyle integration, a subdimension of attitude towards sports, positively influences psychological resilience.

*Hypothesis* 3: Active sport participation behavior, a subdimension of attitude towards sports, positively influences psychological resilience.

*Hypothesis* 4: Interest in sports, a subdimension of attitude towards sports, positively influences life engagement.

*Hypothesis* 5: Sport lifestyle integration, a subdimension of attitude towards sports, positively influences life engagement.

*Hypothesis* 6: Active sport participation behavior, a subdimension of attitude towards sports, positively influences life engagement.

## 2 Methods

### 2.1 Research design

The present investigation employed a correlational survey methodology to examine the relationships between study variables. Correlational survey designs represent systematic research approaches designed to identify and quantify the existence and magnitude of covariation patterns between specified variables (36).

### 2.2 Participants

Data collection for this study was carried out between 30 August 2024 and 10 October 2024 through systematic survey administration protocols. The target population initially consisted of 175 individuals actively participating in swimming training programs in Hatay Province. From this initial group, 51 participants were excluded from the analytical sample because their ages fell outside the defined developmental criteria (below 7 years or exceeding 17 years), which were established for the childhood and adolescent phases under investigation. The final analytical sample comprised 124 participants who met all specified inclusion criteria. The mean age of these participants was 12.69 years (SD = 3.34). Regarding gender distribution, the sample included 72 female participants (58.1%) and 52 male participants (41.9%), all of whom were engaged in structured swimming training programs.

### 2.3 Inclusion criteria

Participant eligibility for study enrollment required satisfaction of the following criteria:

1-Voluntary Informed Participation: Participants had to show a complete understanding of the study’s objectives, procedures, and implications by providing informed consent.
2-Geographic Earthquake Exposure: Participants were required to have resided within the seismically affected geographical zones during the earthquake.
3-Developmental Age Parameters: Participants’ ages needed to fall within the specified range of 7 to 17 years, aligning with the targeted childhood and adolescent developmental stages.

### 2.4 Data collection instruments

The research protocol incorporated a thorough battery of psychometric instruments designed to capture demographic characteristics and primary study constructs. A researcher-developed demographic questionnaire systematically collected participant information pertaining to gender classification and chronological age distributions. Beyond these demographic indicators, the investigation employed three previously validated and psychometrically sound standardized assessment instruments, as detailed below:

#### 2.4.1 Attitude towards sports scale

Originally developed by Senturk (37), the scale represents a multidimensional instrument designed to evaluate attitudinal orientations toward athletic engagement. It comprises 25 items distributed across three theoretically derived subdimensions: (a) Interest in Sports, (b) Sport Lifestyle Integration, and (c) Active Sport Participation Behavior. Response format utilizes a five-point Likert-type rating scale enabling nuanced attitudinal assessment.

#### 2.4.2 Child and youth resilience measure

The scale was originally conceptualized by Liebenberg et al. (38) and subsequently adapted for Turkish populations by Arslan (39) the scale constitutes a unidimensional instrument designed to evaluate psychological resilience capacity among developmental populations. The scale incorporates 12 systematically developed items utilizing a five-point Likert-type response format for in-depth resilience assessment.

#### 2.4.3 Life engagement scale

Originally developed by Scheier et al. (32) and adapted for Turkish linguistic and cultural contexts by Ugur and Akın (40), the LES is a unidimensional psychometric instrument designed to evaluate life engagement levels. The scale comprises six systematically constructed items employing a five-point Likert-type rating format.

### 2.5 Statistical analysis

The analytical framework commenced with data screening protocols examining missing value patterns followed by systematic outlier identification and assessment. Distributional normality evaluation focused on two fundamental parameters: kurtosis and skewness coefficients. In order to satisfy normal distribution assumptions, established psychometric criteria require kurtosis and skewness values to fall within the acceptable range of −1. to +1. Examination of distributional parameters within the current dataset confirmed adherence to normality assumptions across all measurement variables. Internal consistency reliability assessment utilized Cronbach’s Alpha (*α*) coefficients to evaluate the psychometric integrity of measurement instruments. The obtained reliability indices demonstrated acceptable levels of internal consistency, meeting established psychometric standards for research applications (41). There are also opinions that the values of skewness and kurtosis can take values between −2 and +2 (42). Scale scoring procedures employed mean scores derived from constituent items for each measurement instrument. The statistical analysis protocol incorporated descriptive statistical procedures supplemented by inferential techniques including Pearson product–moment correlation analysis and multiple regression modeling. Prior to regression analysis implementation, assumption testing was performed to verify: (a) residual normality distributions, (b) linear relationship characteristics between predictor and criterion variables, (c) homoscedasticity of error variance, (d) independence of observational units, and (e) absence of multicollinearity effects and influential outlier impacts on model parameters. All statistical analyses were conducted at a 95% confidence interval framework, corresponding to an alpha level less than 0.05 (*p* < 0.05) for significance testing protocols.

## 3 Results

Psychometric analysis within the current study demonstrated acceptable internal consistency reliability coefficients: Interest in Sports (*α* = 0.84), Sport Lifestyle Integration (α = 0.72), and Active Sport Participation Behavior (α = 0.75; Table 1). Distributional characteristics analysis showed the following normality values for these sub-dimensions: Interest in Sports (Skewness = −0.520, Kurtosis = −0.192); Sport Lifestyle Integration (Skewness = −0.069, Kurtosis = −0.385); and Active Sport Participation Behavior (Skewness = −0.379, Kurtosis = 0.222). Psychological resilience for internal consistency reliability analysis within the present sample yielded a Cronbach’s Alpha coefficient of *α* = 0.72, indicating acceptable psychometric properties. Distributional normality assessment validated skewness (−0.867) and kurtosis (1.020) parameters, suggesting adequate distributional characteristics for parametric analytical procedures. Life Engagement for reliability analysis within the current research sample demonstrated acceptable internal consistency with a Cronbach’s alpha coefficient of α = 0.72. Examination of distributional characteristics revealed skewness (0.415) and kurtosis (0.623) values, indicating appropriate normality parameters for subsequent statistical analyses. The psychometric evaluation of measurement instruments revealed generally acceptable internal consistency coefficients, though some subdimensions approached the lower threshold of conventional reliability standards. Specifically, the Sport Lifestyle Integration subdimension demonstrated a Cronbach’s alpha coefficient of 0.72, which, while meeting minimum acceptability criteria (*α* ≥ 0.70), represents a more modest reliability estimate compared to optimal psychometric standards (α ≥ 0.80). This finding may reflect several methodological considerations: (a) potential variability in item interpretation among participants from diverse backgrounds, (b) conceptual heterogeneity within the measured construct, or (c) the complex nature of lifestyle integration concepts in post-disaster populations. Despite these considerations, the overall psychometric properties of the measurement battery, including convergent validity and factorial structure, support the reliability of subsequent statistical analyses. Internal consistency coefficients were assessed using Cronbach’s alpha (α) values, with all instruments demonstrating adequate reliability for research purposes (41, 64).

**Table 1:** Measurement instruments, kurtosis and skewness values, and Cronbach’s Alpha (*α*) values.

Measurement instruments	skewness	kurtosis	Cronbach’s alpha (α)
Interest in sports	−0.520	−0.192	0.84
Sport lifestyle integration	−0.069	−0.385	0.72
Active sport participation behavior	−0.379	0.222	0.75
Psychological resilience	−0.867	1.020	0.72
Life engagement	0.415	0.623	0.72

Correlational analytical procedures examine covariation patterns between variables to quantify the magnitude and direction of bivariate relationships (36). Correlation analyses revealed a significant positive moderate association between interest in sports and psychological resilience (*r* = 0.391), and a significant positive weak association between interest in sports and life engagement (*r* = 0.252); a significant positive weak relationship between sport lifestyle integration and psychological resilience (*r* = 0.242); and a significant positive weak relationship between active sport participation and life engagement (*r* = 0.183) (Table 2).

**Table 2:** Correlation analysis results for research variables.

Variables	1	2	3	4	5
Interest in sports	-				
Sport lifestyle integration	0.716^**^	-			
Active sport participation behavior	0.537^**^	0.667^**^	-		
Psychological resilience	0.391^**^	0.242^**^	0.069	-	
Life engagement	0.252^**^	0.094	0.183^*^	0.112	-
Mean	3.83	3.68	3.36	4.10	3.26
Standard deviation	0.644	0.661	0.731	0.488	0.549

**p < 0.01; *p < 0.05. 1: Interest in Sports, 2: Sport Lifestyle Integration,3: Active Sport Participation Behavior, 4: Psychological resilience, 5: Life engagement.

The initial segment of Table 3 presents multiple regression analysis examining the predictive relationships between sport attitude dimensions (independent variables) and psychological resilience (dependent variable). Multicollinearity assessment verified Variance Inflation Factor (VIF) values below the critical threshold of 5.0, indicating absence of problematic intercorrelation among predictor variables. The Durbin-Watson statistical evaluation demonstrated values within the acceptable range of 1.5–2.5, confirming absence of autocorrelation effects within the regression model. The overall regression model achieved statistical significance (F_(df = 3.120)_ = 8.880; *p* = 0.000), indicating that sport attitude dimensions collectively predict psychological resilience outcomes. The model demonstrated an R^2^ value of 0.182 with an adjusted R^2^ of 0.161, suggesting that sport-oriented attitudes account for approximately 16% of the variance in psychological resilience scores among the sample population. Examination of standardized regression coefficients (*β*) showed that *Interest in Sports* emerged as the sole significant predictor within the model (*β* = 0.473), demonstrating a moderate positive effect on psychological resilience outcomes. This finding provides empirical support for Hypothesis 1 (H1), supported that *Interest in Sports* positively influences psychological resilience among earthquake-affected youth. In the context of the relevant finding, it can be thought that sports-oriented studies should be included in the supportive activities to be carried out after the earthquake, based on the fact that being interested in sports positively affects the psychological resilience of earthquake survivors.

**Table 3:** Research variables multiple regression analysis results.

Model	B	Std. error	Beta (β)	t	*p*	VIF
Part 1						
(Constant)	3.083	0.257	-	11.986	0.000	-
Interest in sports	0.358	0.090	0.473	3.972	0.000^***^	2.078
Sport lifestyle integration	0.036	0.099	0.049	0.364	0.717	2.662
Active sport participation behavior	−0.145	0.074	−0.218	−1.953	0.053	1.826
*Dependent Variable: Psychological Resilience*						
*Model Summary: R = 0.426; R^2^ = 0.182; Adj. R^2^ = 0.161; F(_3–120_) = 8.880; p = 0.000; D-W = 1.859*						
Part 2						
(Constant)	2.503	0.304	-	8.227	0.000	-
Interest in sports	0.305	0.107	0.357	2.855	0.005^**^	2.078
Sport lifestyle integration	−0.233	0.118	−0.281	−1.982	0.050	2.662
Active sport participation behavior	0.134	0.088	0.178	1.521	0.131	1.826
*Dependent Variable: Life Engagement*						
*Model Summary: R = 0.310; R^2^ = 0.096; Adj. R^2^ = 0.074; F(_3–120_) = 4.263; p = 0.007; D-W = 1.638*						

^***^p < 0.001; ^**^p < 0.01; Beta (β), Standardized coefficients; D-W, Durbin Watson; VIF, Variance Inflation Factor.

The second analytical segment of Table 3 presents multiple regression analysis examining predictive relationships between sport attitude dimensions (independent variables) and life engagement (dependent variable). The constructed regression model achieved statistical significance (F_(df = 3.120)_ = 4.263; *p* = 0.007), indicating that sport attitude components collectively predict life engagement outcomes. The model demonstrated an R^2^ value of 0.096 with an adjusted R^2^ of 0.074, suggesting that sport-oriented attitudes explain approximately 7% of the variance in life engagement scores. While representing a smaller effect size compared to the psychological resilience model, this finding nevertheless indicates meaningful predictive relationships. Analysis of standardized regression coefficients demonstrated that *Interest in Sports* constituted the primary significant predictor (*β* = 0.357), demonstrating a weak-to-moderate positive effect on Life Engagement outcomes. This empirical evidence provides support for Hypothesis 4 (H4), confirming that Interest in Sports positively influences life engagement among disaster-affected pediatric and adolescent populations. Looking at this finding, it can be said that the interest of earthquake survivors in sports positively affects their commitment to life, contributing to making sense of life and establishing bonds again after major disasters.

## 4 Discussion

This study investigated the relationship between attitudes towards sports and both psychological resilience and life engagement in a cohort of earthquake-affected children and adolescents. Our correlation analyses uncovered several significant associations that warrant careful consideration. Significant positive correlations were detected between the Interest in Sports subdimension and both psychological resilience and life engagement. Another significant but modest positive correlation emerged between Sport Lifestyle Integration and psychological resilience, while Active Sport Participation Behavior demonstrated a significant positive correlation with life engagement.

The regression models established in this investigation demonstrated meaningful explanatory power regarding the relationships under examination. Attitude toward sports accounted for 16% of the variance in psychological resilience scores, with the Interest in Sports subdimension serving as the sole significant predictor. This finding provides empirical support for hypothesis H1, which posited that interest in sports exerts a positive influence on psychological resilience among earthquake survivors. Similarly, attitude toward sports explained 7% of the variance in life engagement, with Interest in Sports again emerging as the significant predictive factor. This result corroborates hypothesis H4, which proposed that interest in sports positively influences life engagement in the target population.

Our research findings align with existing literature demonstrating the positive impact of sports participation on life engagement and mental health outcomes (43). In particular, young people’s perceptions and attitudes regarding sporting activities serve as fundamental determinants of their psychological fortitude and overall life engagement when navigating the challenging circumstances in the aftermath of an earthquake. These discoveries emphasize the critical necessity of incorporating structured physical activities and athletic programs into broad recovery and community restoration initiatives targeting disaster-affected populations. Such integration proves particularly valuable for enhancing the psychosocial welfare of children and adolescents during post-seismic recovery phases our observations documented notable improvements in both psychological resilience capacities and life engagement metrics among youth participants who exhibited favorable orientations toward athletic pursuits. Despite the identification of meaningful associations within our data, however, the magnitude of inter-variable relationships remained moderately constrained. This limitation suggests that further investigations spanning broader demographic categories are essential to establish the generalizability of these beneficial outcomes across diverse population segments.

Contemporary scholarship emphasizes the fundamental importance of consistent physical activity integration within daily routines for achieving optimal mental and physical equilibrium. In addition, social and psychological determinants play pivotal roles in shaping individual attitudes toward sporting engagement (44). Similarly, Malm et al. (45) examined public health outcomes in Swedish populations, analyzing both physiological and psychosocial benefits derived from athletic participation and physical activity engagement. Their findings confirmed that regular physical exercise serves protective functions against various health conditions, particularly depressive symptomatology and stress-related disorders. Individual participation in sporting and physical activities correlates with improved psychological well-being, contributing to healthy mental development through self-acceptance motivation, positive relationship establishment, life purpose development, emotional regulation encouragement, and psychological resilience building (46). In a similar, Peng et al. (47) investigated physical exercise effects on adolescent self-efficacy, focusing on psychological resilience’s mediating role. Their research concluded that self-efficacy increased both directly and indirectly through psychological resilience pathways. The study additionally established that physical activity engagement enhanced participant resilience and self-confidence levels.

Sheng et al. (48) examined the relationship between sports participation and mental resilience in their comprehensive study involving 67,281 children and adolescents. They found a positive connection with the mental resilience of those who participated with an interest in sports. In this context, they stated that the strategy of encouraging children and adolescents to sports has great potential. Hu et al. (49) emphasized the importance of psychological resilience and motivation to exercise in their study of 2,588 adolescent participants and found a positive relationship with sports participation. Tang et al. (50) have addressed the aspect of psychological resilience in adolescents that increases sports participation along with physical self-esteem, and stated the contribution of social support, especially with the motivation to exercise. Zhang and Li (51) found the positive impact of a positive sports environment on psychological resilience in a study involving 315 university students. As seen in the relevant studies, inferences were reached in parallel with our findings in the relationship between sports and psychological resilience.

Han and Polat (52) investigated the relationship between sports commitment and life satisfaction, and in this study, they examined 255 adults. In the relevant study, they mentioned the high positive relationship between sports commitment and life satisfaction. Han et al. (53) found the relationship between sports life satisfaction and life commitment. They mentioned that with the increase in satisfaction with sports life, commitment to life also increases. Selviani et al. (54) examined the reflections of sports participation on life, pointing out that sports have aspects that energize life and lead individuals to spiritual well-being. As can be seen in the research examples in the literature, the positive relationship between sports and life commitment is similar to our findings.

Longitudinal disaster recovery research provides compelling evidence for physical activity’s restorative value. Tian et al. (55) conducted a three-year follow-up study examining post-traumatic stress disorder manifestations in 4604 students following the devastating 8.0 magnitude Wenchuan earthquake. Their investigation emphasized the critical importance of physical exercise and social support systems, advocating for professional, evidence-based interventions targeting students deprived of physical activity opportunities. Wang et al. (56) explored physical activity and life event impacts on university students’ psychological functioning through psychological vulnerability mediation analysis. Their research demonstrated that physical activity engagement supports participants’ psychological states, which typically suffer adverse effects following significant life events. The investigators concluded that consistent physical activity participation can mitigate psychological vulnerability while simultaneously enhancing psychological resilience capacities.

In their study with 434 sports sciences faculty students who experienced the earthquake after the Kahramanmaraş earthquakes, Atılgan and Öztürk (5) investigated the participants’ knowledge about the earthquake and the possible trauma situations they experienced after the earthquake. Information was collected from the students on the relevant subject at two different times. In their findings, they found that the earthquake knowledge of the participants was low and the trauma level was high in the first stage close to the earthquake. In the second stage, they mentioned that there was an increase in the level of knowledge about the earthquake and a decrease in the levels of trauma. The positive change in sports science students over time brings to mind the possible protective aspect of sports education and exercise activities. In their study examining the reflections of the Kahramanmaraş earthquake on social media, Kaya Kılıç et al. (11) drew attention to the high number of calls for help for the repair of their mental aspects immediately after posts in which people demanded basic living materials such as food, water, and clothing. Kurt et al. (57), in their study with parents after the Kahramanmaraş earthquake, mentioned that children showed many emotional and behavioral problems and emphasized the need to resort to special intervention methods since they are in the high-risk group. Balanlı et al. (15) examined their motivations for their participation in physical activity after an earthquake in their study with 276 adolescent participants. In the research findings, it was determined that men had less motivation, while in terms of sports type, participants who did team sports had higher motivation. In their study on physical education and sports teachers affected by earthquakes, Yiğit et al. (58) examined the relationship between the psychological resilience of the participants and earthquake traumas. In the research, which also included life commitment and coping style, they stated that psychological resilience increases life commitment and reduces the effects of trauma. Çakır et al. (10) examined the stress and trauma status of participants who did and did not do sports in their research with 678 university students after the Kahramanmaraş earthquake. Among their findings, they stated that there was a positive difference in the coping of stress and trauma in the group that did sports compared to those who did not. Similarly, related studies by Murathan and Alan (59) and Hazar et al. (60) also drew attention to the healing power of sports after earthquakes. As seen in the latest examples in the earthquake-related literature, it is seen that sports contributions are positive in the evolution of the traumatic situation after the earthquake.

In studies on the post-earthquake traumatic situation and sports-related variables, priority groups in terms of risk are included, and research on groups that are thought to be disadvantaged is increasing. In addition, it is seen that they draw attention to the importance of early intervention in these studies. Studies recommend diversifying classical psychological and social supports and renewing them by adding components such as sports. On the other hand, it is thought that there is not enough diversity in the concrete suggestions for practical applications of the researches recommending the addition of the sports component in post-earthquake recovery studies.

Athletic activities serve as powerful catalysts for social processes that facilitate individual adaptation following catastrophic events such as seismic disasters, simultaneously encouraging community bond reestablishment (61). This social dimension of sporting engagement proves particularly valuable in post-disaster contexts where community cohesion requires rebuilding. Guinto and Logan (22) conducted focused research with 39 typhoon survivors in the Philippines, examining sporting activities’ contribution to recovery processes. Their findings implied that athletic engagement and associated social processes could significantly improve participants’ emotional recovery and psychological rehabilitation trajectories, once again underscoring the dual benefits of sporting activities: direct psychological benefits and indirect social support mechanisms.

## 5 Conclusion and recommendations

This study explored the complex interplay between attitudes toward sports, psychological resilience, and life engagement in children and adolescents affected by an earthquake. Our findings demonstrate significant positive correlations: specifically, Interest in Sports correlated positively with both psychological resilience and life engagement. Furthermore, Sport Lifestyle Integration showed a positive link to psychological resilience, while Active Sport Participation Behavior was positively associated with life engagement. Multiple regression analyses provided confirmatory evidence that sporting interest exerts beneficial influences on both psychological resilience and life engagement parameters. In the light of the information obtained as a result of this research, the relationship between the variables of interest in sports, psychological resilience and commitment to life was determined for the first time in the case of young earthquake survivors. It is thought that being interested in sports is valuable both in terms of the need for psychological resilience in overcoming the negative mental reflections of the earthquake and the contribution of being interested in sports to gaining the motivation to commit to life again after the difficult situation.

Despite the significant findings, the magnitude of observed relationships between sporting attitudes and psychological resilience (16%) and life engagement (7%) remained comparatively moderate. These findings suggest that while cultivating favorable sporting attitudes represents a valuable element within post-earthquake recovery efforts, such approaches cannot function as blanket standalone interventions. Instead, sporting attitude enhancement should be strategically integrated within multifaceted frameworks encompassing diverse psychosocial support modalities. The documented positive associations between sporting attitudes and enhanced resilience and life engagement underscore the remedial potential of physical activity engagement for pediatric and adolescent disaster survivors.

To expand upon these findings, future research should explore the effects of attitudes toward sports across diverse age groups to understand the full breadth of its impact. Moving beyond cross-sectional studies, we highly recommend conducting inclusive longitudinal studies to track the sustained, long-term contributions of sports programs to recovery and the return to normalcy following disasters. Furthermore, researchers should investigate the efficacy of various sports and recreational activities integrated into the recovery process. Finally, adopting mixed-methods research designs, which integrate both quantitative and qualitative approaches, would provide a better understanding of the multifaceted interplay between sports engagement and post-disaster well-being. Continued and diversified research in these areas will significantly contribute to developing evidence-based approaches that can inform policies for disadvantaged groups, particularly children and adolescents, in disaster-affected communities.

The current study’s limitations are primarily rooted in its sampling methodology and restricted scope. Our use of convenience sampling confined the participant pool to 124 earthquake-affected children and adolescents engaged in regular swimming training within a single province. This methodological choice inherently limits the generalizability of our findings to broader populations of youth impacted by earthquakes or to those participating in different sports. On the other hand, it is thought that it is important that this sample group is among the first findings in the field that was reached in a feasible and rapid manner, considering the chaotic and sensitive field conditions of the post-earthquake period.

## Data Availability

The raw data supporting the conclusions of this article will be made available by the authors, without undue reservation.
