# Correction: Attitude towards sports, psychological resilience and life engagement: a study on earthquake victims

**DOI:** 10.3389/fpubh.2026.1927750

**Published:** 2026-09-11

**Authors:** Tolga Tek, Arif Özsari, Murat Tilki, Ṣekip Can Deli, M. Çağri Çetin

**Affiliations:** 1 Faculty of Sport Sciences, Selcuk University, Konya, Türkiye; 2 Faculty of Sport Sciences, Mersin University, Mersin, Türkiye; 3 Institute of Social Sciences, Mus Alparslan University, Mus, Türkiye

**Keywords:** attitude towards sports, earthquake victims, life engagement, psychological resilience, sports

In the published article, there was an error in the wording used to describe the strength of some correlations in the **Abstract *(Result)***:

*Result:* Correlation analyses revealed significant positive moderate associations between interest in sports and psychological resilience (*r* = 0.391) and life engagement (*r* = 0.252); significant relationships between sport lifestyle integration and psychological resilience (*r* = 0.242); and meaningful connections between active sport participation and life engagement (*r* = 0.183). Multiple regression analyses demonstrated that interest in sports significantly predicted both psychological resilience (β = 0.473) and life engagement (β = 0.357).

This has been corrected to read:

“**Result:** Correlation analyses revealed a significant positive moderate association between interest in sports and psychological resilience (*r* = 0.391), and a significant positive weak association between interest in sports and life engagement (*r* = 0.252); a significant positive weak relationship between sport lifestyle integration and psychological resilience (*r* = 0.242); and a significant positive weak relationship between active sport participation and life engagement (*r* = 0.183). Multiple regression analyses demonstrated that interest in sports significantly predicted both psychological resilience (β = 0.473) and life engagement (β = 0.357).”

In the published article, there was an error in the wording used to describe the strength of some correlations in the **Results:**

Results: Correlational analytical procedures examine covariation patterns between variables to quantify the magnitude and direction of bivariate relationships (36). Analyses showed significant positive associations between sport attitude dimensions and psychological outcome variables. The interest in sports subdimension demonstrated moderate positive correlations with both psychological resilience capacity (*r* = 0.391) and life engagement (*r* = 0.252). The *Sport Lifestyle Integration* subdimension exhibited a weak-to-moderate positive correlation with psychological resilience (*r* = 0.242). Besides, the *Active Sport Participation Behavior* dimension manifested a weak but statistically significant positive association with life engagement (*r* = 0.183) (Table 2).

This has been corrected to read:

“**Results:** Correlational analytical procedures examine covariation patterns between variables to quantify the magnitude and direction of bivariate relationships (36). Correlation analyses revealed a significant positive moderate association between interest in sports and psychological resilience (*r* = 0.391), and a significant positive weak association between interest in sports and life engagement (*r* = 0.252); a significant positive weak relationship between sport lifestyle integration and psychological resilience (*r* = 0.242); and a significant positive weak relationship between active sport participation and life engagement (*r* = 0.183) (Table 2).”

The original version of this article has been updated.

